# Effects of nucleases on cell-free extrachromosomal circular DNA

**DOI:** 10.1172/jci.insight.156070

**Published:** 2022-04-22

**Authors:** Sarah T.K. Sin, Jiaen Deng, Lu Ji, Masashi Yukawa, Rebecca W.Y. Chan, Stefano Volpi, Augusto Vaglio, Paride Fenaroli, Paola Bocca, Suk Hang Cheng, Danny K.L. Wong, Kathy O. Lui, Peiyong Jiang, K.C. Allen Chan, Rossa W.K. Chiu, Y.M. Dennis Lo

**Affiliations:** 1 Li Ka Shing Institute of Health Sciences and; 2 Department of Chemical Pathology, Prince of Wales Hospital, the Chinese University of Hong Kong, Shatin, New Territories, Hong Kong SAR, China.; 3 Centre for Novostics, Hong Kong Science Park, the Chinese University of Hong Kong, Pak Shek Kok, New Territories, Hong Kong SAR, China.; 4 Pediatric and Rheumatology Clinic, Center of Autoinflammatory Diseases and Immunodeficiencies, Scientific Hospitalization and Treatment Institute (IRCCS), Giannina Gaslini Institute, Genova, Italy.; 5 Department of Neuroscience, Rehabilitation, Ophthalmology, Genetics and Maternal-Child Sciences (DINOGMI), University of Genova, Genova, Italy.; 6 Department of Experimental and Clinical Biomedical Sciences “Mario Serio,” School of Human Health Sciences, University of Florence, Florence, Italy.; 7 Medical Genetics Unit and; 8 Nephrology and Dialysis Unit, Meyer Children’s Hospital, Florence, Italy.; 9 Nephrology Unit, University Hospital of Parma, Parma, Italy.

**Keywords:** Genetics, Genetic diseases, Molecular diagnosis, Mouse models

## Abstract

Cell-free extrachromosomal circular DNA (eccDNA) as a distinct topological form from linear DNA has recently gained increasing research interest, with possible clinical applications as a class of biomarkers. In this study, we aimed to explore the relationship between nucleases and eccDNA characteristics in plasma. By using knockout mouse models with deficiencies in deoxyribonuclease 1 (DNASE1) or deoxyribonuclease 1 like 3 (DNASE1L3), we found that cell-free eccDNA in *Dnase1l3*^−/−^ mice exhibited larger size distributions than that in wild-type mice. Such size alterations were not found in tissue eccDNA of either *Dnase1*^−/−^ or *Dnase1l3*^−/−^ mice, suggesting that DNASE1L3 could digest eccDNA extracellularly but did not seem to affect intracellular eccDNA. Using a mouse pregnancy model, we observed that in *Dnase1l3*^−/−^ mice pregnant with *Dnase1l3*^+/−^ fetuses, the eccDNA in the maternal plasma was shorter compared with that of *Dnase1l3*^−/−^ mice carrying *Dnase1l3*^−/−^ fetuses, highlighting the systemic effects of circulating fetal DNASE1L3 degrading the maternal eccDNA extracellularly. Furthermore, plasma eccDNA in patients with *DNASE1L3* mutations also exhibited longer size distributions than that in healthy controls. Taken together, this study provided a hitherto missing link between nuclease activity and the biological manifestations of eccDNA in plasma, paving the way for future biomarker development of this special form of DNA molecules.

## Introduction

Cell-free DNA (cfDNA) molecules are present in plasma in either linear or circular forms (1, 2). In addition to the finding that a subset of mitochondrial genomes could exist as circular forms in plasma depending on their tissues of origin (3), cell-free extrachromosomal circular DNA (eccDNA) was detectable in plasma of pregnant women, healthy participants, and patients with cancer, albeit at lower abundance than their linear counterparts (4–6). Larger eccDNA molecules of megabase sizes have been shown to be enriched in cancer cells harboring parts of or whole oncogene sequences, conferring possible advantages in proliferation and drug resistance of these cells (7–9). In addition, eccDNA molecules have been shown to accumulate in aging cells, providing extra genetic heterogeneity for those cells to rapidly adapt to environmental stressors (10–12). Interestingly, by transfecting eccDNA into bone marrow–derived dendritic cells, Wang et al. demonstrated that eccDNA could function as a potent immunostimulant (13).

In contrast to linear cfDNA with 1 predominant peak at 166 bp, size profiles of eccDNA in plasma exhibited 2 major peak clusters with summits at around 202 and 338 bp, along with a series of sharp 10 bp periodic subpeaks within both clusters. Such characteristic size profiles of eccDNA suggested possible involvements of nucleosomal structures (4). Interestingly, fetal-specific eccDNA molecules were reported to be detectable in the maternal plasma of pregnant women and were shorter and less methylated than the maternal ones (4, 14). Therefore, the biological properties of eccDNA molecules might depend on their tissues of origin.

The fragmentation of linear cfDNA is a nonrandom process. Multiple lines of evidence suggest that such fragmentation patterns could be linked to the activity of various nucleases (15). For instance, it has been reported that deoxyribonuclease 1 like 3 (DNASE1L3) contributes to cfDNA fragmentation and preferentially generates fragments with CC ends both in mice and in humans (16, 17). Han et al. systematically studied the effects of DNASE1, DNASE1L3, and DNA fragmentation factor subunit β (DFFB) on cfDNA fragmentation and found that these nucleases act on DNA degradation during cell apoptosis in a stepwise manner (18). In addition, fragmented cfDNA was undetectable in mice with double deletion of *Dnase1l3* and *Dffb* (19). Given such effects of nucleases on linear cfDNA, we postulated that certain nucleases might also play roles in the generation and/or degradation of eccDNA in plasma.

In this study, we used knockout mouse models to explore whether nucleases such as DNASE1L3 and DNASE1 would affect the biological properties of plasma eccDNA. By analyzing the eccDNA size patterns of plasma and tissue eccDNA in mice deficient in either nuclease, we tried to decipher whether these nucleases act on eccDNA in intracellular or extracellular manners. Furthermore, using a mouse pregnancy model, we examined the effects of extracellular DNASE1L3 on cell-free eccDNA. Further evidence of nuclease effects on eccDNA in humans was provided by comparing the cell-free eccDNA profiles between patients with *DNASE1L3* mutations and healthy controls.

## Results

### Study design.

**Figure 1:**
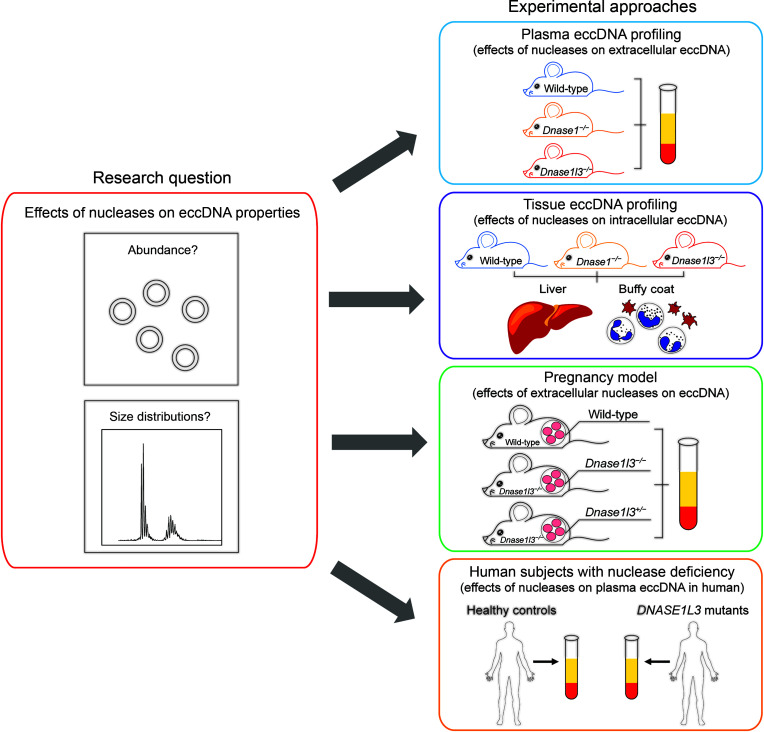
Study design. Experimental approaches were designed to explore whether nucleases (DNASE1 and DNASE1L3) would have any effects on eccDNA characteristics. First, knockout mouse models with deficiencies in *Dnase1* or *Dnase1l3* were used to investigate whether eccDNA attributes such as abundance and size distribution were altered when compared with wild-type mice. Such comparisons were performed in both plasma and tissue (the liver and buffy coat) eccDNA among the 3 groups of mice to elucidate whether the nuclease effects on eccDNA, if any, were exerted extracellularly or intracellularly. Subsequently, a *Dnase1l3*^−/−^ mouse pregnancy model was used to determine whether extracellular DNASE1L3 the fetuses released would act on the eccDNA molecules in maternal plasma, altering their size distributions. Furthermore, DNASE1L3’s effects on cell-free eccDNA were tested in human participants: size distributions of eccDNA from plasma samples were compared between healthy participants and patients with *DNASE1L3* mutations.

Figure 1 illustrates the conceptual design of this study. We investigated whether nucleases (e.g., DNASE1 and DNASE1L3) would have any effects on eccDNA characteristics of quantity and size distribution. First, we identified the plasma eccDNA molecules from wild-type, *Dnase1*^−/−^, and *Dnase1l3*^−/−^ mice. Normalized counts and size profiles of eccDNA were compared among these 3 groups of mice. Second, to explore whether nucleases act on eccDNA molecules inside live cells, we profiled the cellular eccDNA from the 3 groups of mice in 2 tissue types: the liver and buffy coat. To further interrogate the extracellular effects of nucleases on eccDNA, we applied a pregnancy model to examine whether DNASE1L3 released by the heterozygous (*Dnase1l3*^+/−^) fetuses would have any impacts on the cell-free eccDNA in maternal plasma. Last, we further evaluated nuclease effects on cell-free eccDNA in human patients by comparing eccDNA characteristics between healthy controls and patients with *DNASE1L3* loss-of-function mutations.

### EccDNA size distributions in mouse plasma.

**Figure 2:**
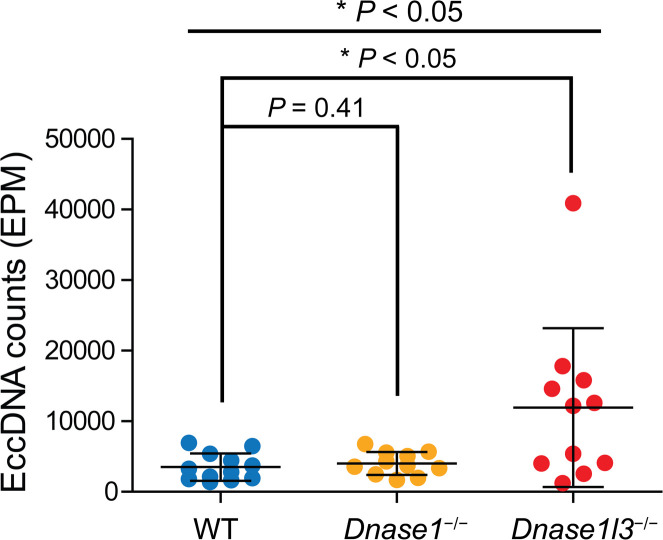
EccDNA counts in the plasma DNA of wild-type, *Dnase1*^−/−^, and *Dnase1l3*^−/−^ mice. To examine whether deficiencies of *Dnase1* or *Dnase1l3* would alter the abundance of cell-free eccDNA in mice, we prepared eccDNA sequence libraries from 12 wild-type, 11 *Dnase1*^−/−^, and 11 *Dnase1l3*^−/−^ mice. Total numbers of eccDNA molecules identified from these mice were normalized to the numbers of mappable reads in each sample and denoted as eccDNA per million mappable reads (EPM) values. Kruskal-Wallis test (*P* < 0.05) followed by Dunn’s multiple-comparison test detected significantly higher EPM values in *Dnase1l3*^−/−^ mice than in wild-type mice.

We sequenced plasma eccDNA libraries with a median of 17,463,304 paired-end reads (range: 11,845,852–27,836,098) using the tagmentation-based eccDNA library preparation protocol as previously described (4) and identified a median of 15,337 eccDNA loci (range: 3309–94,248). More details of the sequencing results are listed in Supplemental Table 1; supplemental material available online with this article; https://doi.org/10.1172/jci.insight.156070DS1 Figure 2 shows the numbers of plasma eccDNA molecules identified among the 3 groups of mice, which were normalized by the number of mappable reads for each sample, denoted as eccDNA per million mappable reads (EPM) values. The EPM values of *Dnase1l3*^−/−^ mice were significantly higher (median: 12,206; range: 1241–40,897) than those of wild-type mice (median: 3056; range: 1404–6952) (*P* = 0.04, Kruskal-Wallis test). No statistically significant difference was observed between wild-type and *Dnase1*^−/−^ mice.

**Figure 3:**
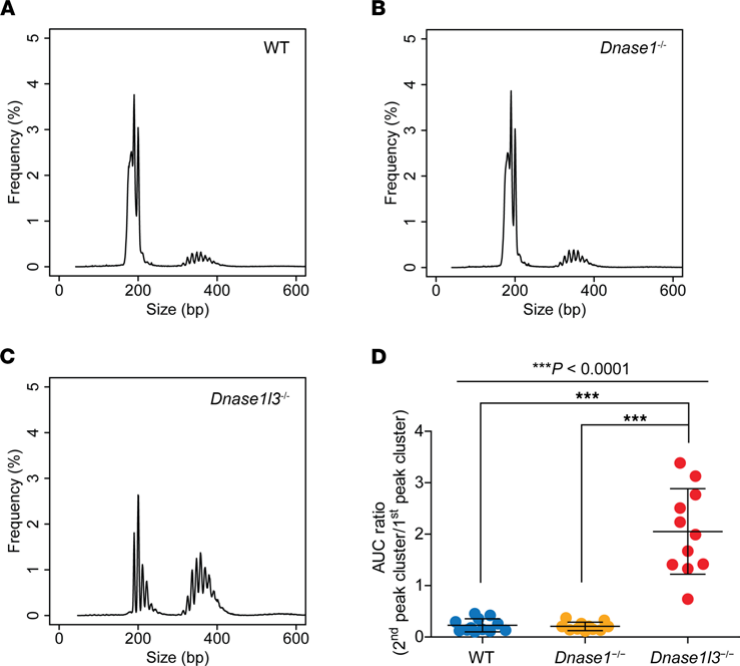
Plasma eccDNA size profiling of wild-type, *Dnase1*^−/−^, and *Dnase1l3*^−/−^ mice. Plasma DNA samples were analyzed for 12 wild-type, 11 *Dnase1*^−/−^, and 11 *Dnase1l3*^−/−^ mice. Data were pooled for each genotype of mice for demonstration of size profiles. (**A**) Wild-type, (**B**) *Dnase1*^−/−^, and (**C**) *Dnase1l3*^−/−^ mice all showed 2 predominant peak clusters with summits at around 200 bp (first peak cluster) and 350 bp (second peak cluster). (**D**) Area under the size profile curve (AUC) ratios of individual mice. Kruskal-Wallis test (*P* < 0.0001) followed by Dunn’s multiple-comparison test showed that *Dnase1l3*^−/−^ mice had significantly higher AUC ratios than wild-type and *Dnase1*^−/−^ mice.

Plasma eccDNA molecules were pooled into 3 groups according to the mouse genotypes. Their size frequencies are plotted in Figure 3, A–C. Plasma eccDNA from all 3 groups of mice showed bimodal size distributions with summits at around 200 bp (first peak cluster) and 350 bp (second peak cluster). Compared with wild-type mice, *Dnase1l3*^−/−^ mice showed a reduction of the first peak cluster (150–250 bp) and an enhancement of the second peak cluster (300–450 bp). However, no such difference was observed between *Dnase1*^−/−^ and wild-type mice. We further calculated the values of area under the size profile curve (AUC) for the 2 peak clusters of the 3 groups of mice. The AUC values of individual mice are plotted in Supplemental Figure 1 and showed that the reduction of the first peak cluster and the enhancement of the second peak cluster of *Dnase1l3*^−/−^ mice (AUC_1st_ _peak_ _cluster_: median 30.3%, range 19.0%–54.6%; AUC_2nd peak_ _cluster_: median 58.9%, range 40.4%–67.7%) were statistically significant in comparison with the wild-type mice (AUC_1st_ _peak_ _cluster_: median 77.9%, range 62.7%–88.1%; AUC_2nd_ _peak_ _cluster_: median 12.8%, range 8.9%–28.5%) (*P*_1st peak_ _cluster_ < 0.0001, *P*_2nd_ _peak_ _cluster_ < 0.0001; Kruskal-Wallis test). To better record the overall size distribution features of eccDNA, we further introduced the parameter of AUC ratio, which was calculated as the AUC of the second peak cluster divided by the AUC of the first peak cluster. Thus, the higher the AUC ratio, the longer the overall sizes of eccDNA. Figure 3D plots the AUC ratios (second peak cluster/first peak cluster) of the 3 groups of mice, which showed that *Dnase1l3*^−/−^ mice had significantly higher AUC ratios than wild-type and *Dnase1*^−/−^ mice. In summary, these data indicated that the plasma eccDNA molecules of *Dnase1l3*^−/−^ mice were, in general, longer than those of wild-type and *Dnase1*^−/−^ mice.

### Sequence contexts of cell-free eccDNA in mouse plasma.

To further explore the genomic distributions of the eccDNA identified in the mouse plasma samples, we tried to associate these molecules with various genomic elements, including 5′ untranslated regions (UTRs), 3′ UTR, gene body regions, CpG islands, and *Alu* repeats (Supplemental Figure 2). EccDNA from the 3 genotypes of mice was enriched in gene body regions because its fold changes relative to expected frequencies (details for the calculation of expected frequencies are described in Methods) were greater than 1, which was consistent with previous studies (4, 6, 20). The eccDNA distributions between the wild-type and the *Dnase1*^−/−^ mice were generally comparable. However, those from the *Dnase1l3*^−/−^ mice showed 53% and 67% increases in 5′ UTR and CpG island regions, respectively, when compared with the wild-type mice. We also compared the eccDNA loci between wild-type and *Dnase1l3*^−/−^ mice. In total, we detected 246,855 and 371,816 cell-free eccDNA loci from wild-type and *Dnase1l3*^−/−^ mice, respectively. Among these loci, only 603 (0.24% of wild-type and 0.16% of *Dnase1l3*^−/−^) were shared between the 2 groups of mice. Therefore, it was rare for eccDNA to be generated from the same loci between the 2 groups of mice.

We previously observed that in human plasma, eccDNA showed repeats of trinucleotide motifs (denoted as motifs I, II, III, and IV) flanking the eccDNA junctions (4). We looked at the trinucleotide junctional motifs of wild-type and *Dnase1l3*^−/−^ mice in this light and compared these motif sequences between the 2 groups. The top 5 combinations of trinucleotide motifs flanking the eccDNA junctions of wild-type and *Dnase1l3*^−/−^ mice are listed in Supplemental Tables 2 and 3, respectively. Since the 2 groups of mice shared 4 out of the top 5 trinucleotide motif combinations, we did not observe obvious differences in junctional motifs between the 2 groups of mice in general.

### EccDNA analyses in mouse tissues.

To explore whether the above-described size differences of plasma eccDNA among different genotypes of mice occurred intracellularly or extracellularly, we profiled the eccDNA extracted from the liver and buffy coat from wild-type, *Dnase1*^−/−^, and *Dnase1l3*^−/−^ mice. Our choices of mouse tissues for eccDNA analyses were for 2 main reasons. First, it has been previously established that white blood cells are major sources of linear cfDNA in the blood circulation, followed by the liver (21, 22). Second, according to publicly available RNA-sequencing data sets, the liver is one of the tissue types with the highest *Dnase1l3* expression levels in mice (23, 24). Thus, we believed that these 2 tissue types would be a good starting point for elucidating the possible mechanisms of eccDNA metabolism from tissue to blood. We applied 2 approaches for tissue eccDNA identification in parallel: the tagmentation-based approach and the rolling circle amplification–based (RCA-based) approach (details described in Methods and illustrated in Supplemental Figure 3A). We compared the eccDNA loci identified from the 2 methods using buffy coat tissues as an example. In the buffy coat of wild-type mice, we identified 34,376 and 128,351 eccDNA loci using tagmentation and RCA, respectively. Among these loci, only 68 (0.2% from tagmentation and 0.05% from RCA) were shared between the 2 methods. In the buffy coat of *Dnase1l3*^−/−^ mice, we identified 13,941 and 55,280 eccDNA loci using tagmentation and RCA, respectively. Among these loci, only 12 (0.09% from tagmentation and 0.02% from RCA) were shared between the 2 methods.

**Figure 4:**
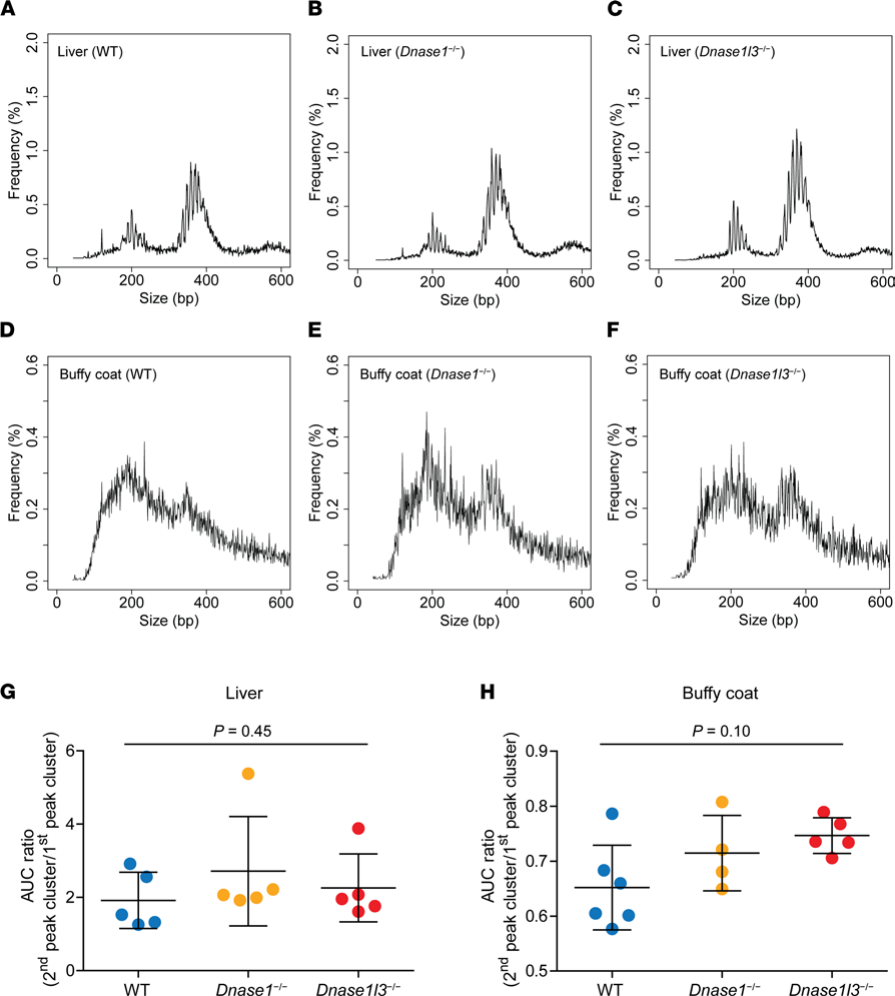
Tissue eccDNA size profiling of wild-type, *Dnase1*^−/−^, and *Dnase1l3*^−/−^ mice using the tagmentation-based method. Liver eccDNA was analyzed in (**A**) 5 wild-type, (**B**) 5 *Dnase1*^−/−^, and (**C**) 5 *Dnase1l3*^−/−^ mice; buffy coat eccDNA was analyzed in (**D**) 6 wild-type, (**E**) 4 *Dnase1*^−/−^, and (**F**) 5 *Dnase1l3*^−/−^ mice. EccDNA identified in each tissue type was pooled for each genotype of mice and size profiled. The AUC ratios were compared among the 3 groups of mice for both (**G**) the liver and (**H**) buffy coat. Kruskal-Wallis test detected no statistically significant difference among the 3 groups of mice in either the liver (*P* = 0.45) or buffy coat (*P* = 0.10).

Tissue eccDNA molecules were pooled for size profiling according to the mouse genotypes and tissue types (tagmentation: Figure 4, A–F; RCA: Supplemental Figure 3, B–G). Individual size profiles are plotted in different colors in Supplemental Figure 4. For the tagmentation approach, we identified eccDNA loci with medians of 3051 (range: 1633–29,176) and 4217 (range: 1952–10,034) from the liver and buffy coat tissues, respectively. For the RCA approach, medians of 10,402 (range: 4355–42,473) and 12,490 (range: 6260–43,288) eccDNA loci were identified from the liver and buffy coat tissues, respectively. The eccDNA molecules originating from these tissues all displayed bimodal size distributions with the 2 summits at around 200 bp and 350 bp. Of note, the 2 peak clusters of the liver eccDNA were sharper than those of the buffy coat eccDNA. The 10 bp periodic oscillations were apparent in the liver eccDNA (reminiscent of plasma eccDNA patterns) but relatively obscure in the buffy coat eccDNA. Such variations hinted that the characteristics of eccDNA might depend on their tissues of origin. To further explore the tissue specificity of eccDNA size distributions, we accessed publicly available data sets from Assay for Transposase-Accessible Chromatin using sequencing (ATAC-seq) (25). We could identify eccDNA sequences from ATAC-seq data sets because tagmentation is an essential step in the ATAC-seq experiments, which would theoretically also capture eccDNA in the DNA library (26). For better representation of size profiles, we included only tissue types with at least 3000 eccDNA loci identified, including spleen (8258 loci), thymus (3965 loci), small intestines (5006 loci), and kidney (4693 loci). EccDNA size profiles of these tissues are shown in Supplemental Figure 5. These 4 tissue types all exhibited a predominant peak cluster at around 200 bp, which was in contrast to the noisy peak signals of mouse buffy coat from our data. Therefore, these ATAC-seq data might provide additional support to the hypothesis that eccDNA size distributions would to a certain degree depend on their tissues or cell types of origin.

Interestingly, no apparent difference in eccDNA size distributions could be observed among wild-type, *Dnase1*^−/−^, and *Dnase1l3*^−/−^ mice for either the liver or buffy coat using the tagmentation method. As shown in Figure 4G, no statistically significant difference in AUC ratios was detected in the liver eccDNA among the 3 groups of mice (*P* = 0.45, Kruskal-Wallis test). The same conclusion held for the buffy coat samples (*P* = 0.10, Kruskal-Wallis test). These results were further validated using the RCA-based method, where no significant difference in AUC ratios was observed among the 3 groups of mice in either the liver (*P* = 0.93, Kruskal-Wallis test; Supplemental Figure 6A) or buffy coat (*P* = 0.93, Kruskal-Wallis test; Supplemental Figure 6B) tissues. The fact that the eccDNA size differences among genotypes were observed in plasma but not in tissue suggested that the effects of DNASE1L3 on intracellular eccDNA were relatively insignificant. Rather, this enzyme was able to act on eccDNA after these molecules were released into the blood circulation.

### Dnase1l3^−/−^ mouse pregnancy model.

To test the hypothesis that the size differences of eccDNA observed in plasma between wild-type and *Dnase1l3*^−/−^ mice are due to the extracellular DNASE1L3 effect, we used the *Dnase1l3*^−/−^ mouse pregnancy model, in which female mice of the C57BL/6 strain with or without *Dnase1l3* deficiency were crossed with wild-type mice from the BALB/c genomic background. As such, 3 mating groups were generated: (i) wild-type females pregnant with wild-type fetuses, (ii) *Dnase1l3*^−/−^ females pregnant with *Dnase1l3*^−/−^ fetuses, and (iii) *Dnase1l3*^−/−^ females pregnant with *Dnase1l3*^+/−^ fetuses. We also exploited the genomic differences between the C57BL/6 and BALB/c strains to distinguish fetus-specific molecules from those shared by the mother and the fetuses (i.e., shared molecules) (see details in Methods). Information about mouse pregnancy samples and their respective eccDNA fetal fractions in the maternal plasma are listed in Supplemental Table 4. The median fetal eccDNA fraction was 25.8% (range: 16.5%–46.6%).

**Figure 5:**
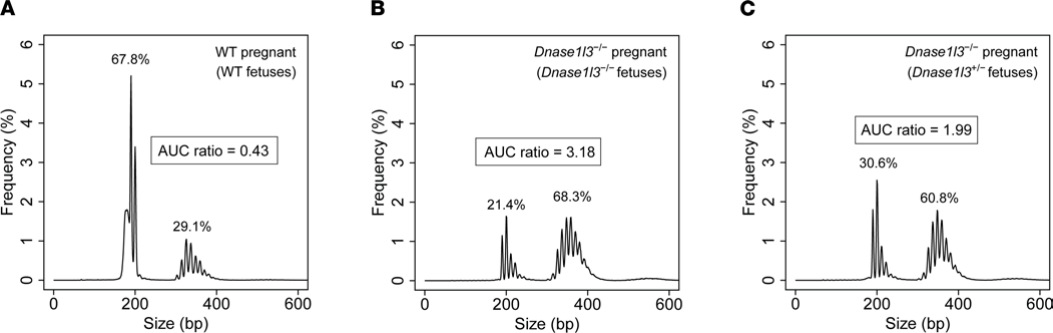
Effects of fetally released DNASE1L3 on cell-free eccDNA in maternal plasma. The mean size distributions of eccDNA in maternal plasma were plotted for (**A**) wild-type females carrying wild-type fetuses, (**B**) *Dnase1l3*^−/−^ females carrying *Dnase1l3*^−/−^ fetuses, and (**C**) *Dnase1l3*^−/−^ females carrying *Dnase1l3*^+/−^ fetuses. AUC values were labeled for each peak cluster, and AUC ratios were calculated accordingly.

Figure 5 plots the mean size distributions of total plasma eccDNA in the 3 groups of pregnant mice. For wild-type females carrying wild-type fetuses (Figure 5A), their plasma eccDNA showed a high first peak cluster (AUC = 67.8%) and a relatively low second peak cluster (AUC = 29.1%), with an AUC ratio of 0.43. On the other hand, for *Dnase1l3*^−/−^ females carrying *Dnase1l3*^−/−^ fetuses (Figure 5B), their plasma eccDNA showed a low first peak cluster (AUC = 21.4%) and a higher second peak cluster (AUC = 68.3%), with an AUC ratio of 3.18. Thus, *Dnase1l3*^−/−^ females pregnant with *Dnase1l3*^−/−^ fetuses had longer plasma eccDNA than wild-type mice carrying wild-type fetuses. This is consistent with the eccDNA size differences observed between the nonpregnant wild-type and *Dnase1l3*^−/−^ mice (Figure 3). However, for the *Dnase1l3*^−/−^ females carrying *Dnase1l3*^+/−^ (heterozygous) fetuses (Figure 5C), their plasma eccDNA sizes showed a partial reversal from the *Dnase1l3*^−/−^ phenotype to the wild-type phenotype, with both AUC values (AUC_1st_ _peak_ _cluster_ = 30.6%; AUC_2nd_ _peak_ _cluster_ = 60.8%) and AUC ratio (1.99) falling between those of the first 2 groups of pregnant mice. These results suggested the presence of systemic effects of fetally released DNASE1L3 preferentially digesting larger eccDNA molecules in maternal circulation, leading to the observation of the higher abundance of smaller eccDNA. Of note, we did not observe the fetal eccDNA being shorter than the maternal eccDNA (shared molecules) in the plasma of *Dnase1l3*^−/−^ mice pregnant with *Dnase1l3*^+/−^ fetuses (Supplemental Figure 7), suggesting that the local effect of DNASE1L3 on eccDNA digestion might not be as significant as it was on linear cfDNA, as previously reported (16). Taken together, the fetally released DNASE1L3 could digest the eccDNA in maternal plasma.

### Human participants with DNASE1L3 deficiency.

**Figure 6:**
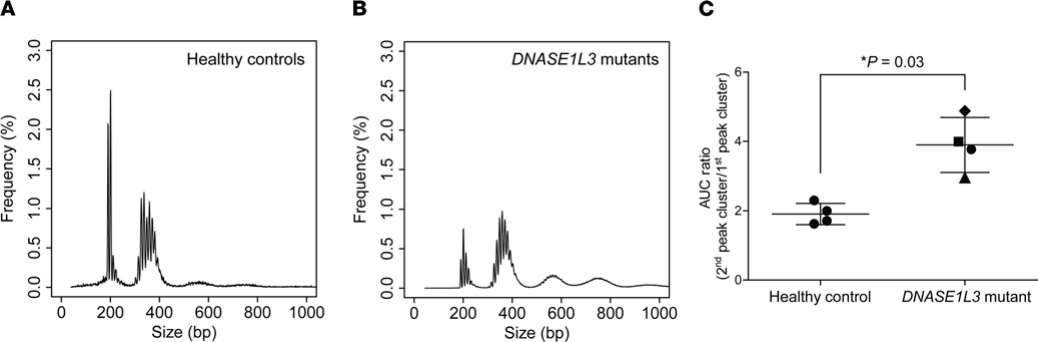
Plasma eccDNA profiling in human participants with *DNASE1L3* mutations. (**A**) Size profile of eccDNA pooled from plasma samples collected from 4 healthy human participants. (**B**) Size profile of eccDNA pooled from 4 plasma samples collected from 3 human patients with loss-of-function mutations in *DNASE1L3*. (**C**) AUC ratios were compared between healthy controls and patients with *DNASE1L3* mutations. *P* = 0.03, Wilcoxon’s rank-sum test. Triangle: prehemodialysis plasma sample from patient P1; square: posthemodialysis plasma sample from patient P1; diamond: plasma sample from patient P2; circle: plasma sample from patient P3.

The effects of *DNASE1L3* deficiency on plasma eccDNA were further investigated in patients with *DNASE1L3* loss-of-function mutations. More information on the clinical characteristics of these patients is in Methods. Sequencing details of the human plasma samples are listed in Supplemental Table 5. We observed a trend of higher eccDNA counts (denoted as EPM values) in the plasma of *DNASE1L3*-mutated human patients compared with the healthy controls (Supplemental Figure 8) similar to *Dnase1l3*-deficient mice. Figure 6, A and B, plot the mean size distributions of plasma eccDNA from healthy human participants and patients with *DNASE1L3* mutations, respectively. When compared with healthy control samples, *DNASE1L3*-mutant samples showed a lower first peak cluster, reflecting a decrease in frequency of small eccDNA in these patients. As shown by the individual size profiles of these patients (Supplemental Figure 9), for the patient who donated blood samples at 2 time points, the eccDNA size distributions were quite similar between the pre- and posthemodialysis time points. Figure 6C presents a comparison of the AUC ratios between healthy controls and patients with *DNASE1L3* mutations: patients with *DNASE1L3* mutations exhibited significantly higher AUC ratios than the healthy controls (*P* = 0.03, Wilcoxon’s rank-sum test). Interestingly, the third and the fourth peak clusters were enriched in the patients with *DNASE1L3* mutations, reflecting a higher abundance of long eccDNA molecules in the plasma of these patients. These additional peak clusters have also been observed in several studies investigating eccDNA in mouse tissues and human cell lines (20, 26). Thus, *DNASE1L3* deficiency in humans would lead to the lengthening of eccDNA size distributions in plasma, consistent with our findings in *Dnase1l3*-knockout mice (Figure 3).

## Discussion

In this study, we demonstrated that the biological properties of eccDNA could be affected by the activity of nucleases. By using knockout mouse models of *Dnase1* and *Dnase1l3*, we found that the deficiency of *Dnase1l3* could markedly lengthen the plasma eccDNA in mice. However, such effects were not observed in mice with *Dnase1* deficiency. We thus believe that DNASE1L3 could be one of the main contributors affecting the size characteristics of cell-free eccDNA. This observation was in agreement with previous findings: DNASE1L3 apparently affects the size distribution of linear cfDNA, whereas DNASE1 contributes less (16, 17, 27).

Intriguingly, the plasma eccDNA size distributions in wild-type mice (Figure 3A) were distinct from those in humans (Figure 6A), with enhanced first peak clusters and diminished second peak clusters. Moreover, the plasma eccDNA size profiles of *Dnase1l3*-deficient mice (Figure 3C) highly resembled those in humans. Such observations might be attributed to the much higher activity of circulating DNASE1L3 present in mice (28). The shortening effect of DNASE1L3 on eccDNA sizes was further highlighted in our data comparing cell-free eccDNA between healthy human participants and patients with *DNASE1L3* mutations (Figure 6). Future analyses of larger patient cohorts might further substantiate our findings in humans regarding DNASE1L3’s effects on cell-free eccDNA. The above lines of evidence consistently suggested that the activity of DNASE1L3 could effectively regulate the biological characteristics of cell-free eccDNA.

Notably, at the intracellular level, neither *Dnase1*^−/−^ mice nor *Dnase1l3*^−/−^ mice showed an observable change in eccDNA size profiles (Figure 4). We thus suspected that the access of intracellular eccDNA by DNASE1L3 is rather limited. Instead, DNASE1L3 could act on eccDNA molecules after they enter the blood circulation. This postulation could in part be supported by the following pieces of evidence on living cells: (i) DNASE1L3 is detected in the endoplasmic reticulum, but absent from the nucleus (29, 30); and (ii) cellular eccDNA is located inside the cell nucleus (20, 31), which would thus limit the access of DNASE1L3 to these molecules. However, the release of eccDNA molecules into the circulation would facilitate their access by DNASE1L3, leading to the degradation of these DNA molecules.

The extracellular function of DNASE1L3 on cell-free eccDNA was further evidenced by our findings from the *Dnase1l3* pregnancy mouse model. It has previously been established that in *Dnase1l3*-deficient mice pregnant with *Dnase1l3*^+/−^ fetuses, DNASE1L3 released from the fetuses could degrade linear cfDNA molecules in a systemic manner (16). Similarly, we observed a partial restoration of eccDNA size profiles toward the wild-type patterns in the maternal plasma under the same pregnancy setting. This finding suggested that the extracellular DNASE1L3 the fetuses produced could act on the eccDNA in the maternal blood circulation, mediating the degradation of maternal cell-free eccDNA. Since DNASE1L3 can be secreted into the blood circulation (28, 30), we believe that similar systemic effects of this nuclease could also be applied to other tissue types when those tissues express *Dnase1l3*. Of note, we did not observe the expected shortening of eccDNA derived from the *Dnase1l3*^+/−^ fetuses when compared to their *Dnase1l3*^−/−^ mothers, which was seemingly in contrast to the previously reported shorter sizes of fetal eccDNA than the maternal molecules in humans (4, 14). Further studies on the interrelationships between maternal and fetal eccDNA metabolism will be needed to reconcile these observations. Although we cannot rule out other possible mechanisms shaping the sizes of cell-free eccDNA, such as its cleavage by intracellular DNASE1L3 in apoptotic cells (32, 33), we conclude from our data that circulating DNASE1L3 functions as an important factor modulating the biological characteristics of eccDNA in plasma.

In summary, we established a biological link between nuclease activity and the properties of cell-free eccDNA using mouse and human models with DNASE1L3 deficiency. It has previously been reported that in patients with *DNASE1L3* mutations, the size profiles and end motif frequencies of linear cfDNA deviate from those of healthy controls (17). In knockout mouse models with *Dnase1l3* deficiency, the methylation profiles and end jaggedness of linear plasma DNA are altered (34, 35). Our findings of DNASE1L3’s effects on cell-free eccDNA characteristics in this study might provide yet another potential biomarker for early diagnosis or monitoring of diseases related to *DNASE1L3* deficiency, such as systemic lupus erythematosus (17, 36).

## Methods

### Mouse sample collection and processing

Blood samples were collected from 12 wild-type, 11 *Dnase1*^−/−^, and 11 *Dnase1l3*^−/−^ mice by cardiac punctures and centrifuged at 1600*g* for 10 minutes at 4°C, followed by another centrifugation step at 16,000*g* for 10 minutes at 4°C of the plasma portion to remove cell debris. The buffy coat portion was centrifuged at 5000*g* for 5 minutes at room temperature to remove residual plasma. Mouse liver tissues were collected and immediately stored at –80°C. Plasma DNA was extracted using QIAamp Circulating Nucleic Acid Kits (QIAGEN). Buffy coat (6 wild-type, 4 *Dnase1*^−/−^, and 5 *Dnase1l3*^−/−^ mice) and liver (5 wild-type, 5 *Dnase1*^−/−^, and 5 *Dnase1l3*^−/−^ mice) tissue DNA was extracted using QIAamp DNA Mini Kits (QIAGEN).

### EccDNA library preparation and sequencing

EccDNA libraries from plasma samples were constructed using the tagmentation-based method as detailed previously (4). For eccDNA enrichment from tissue DNA of the liver and buffy coat, we used a dual-size selection approach using solid-phase reversible immobilization beads (Beckman Coulter). The workflow of this approach is illustrated in Supplemental Figure 3A. Chromosomal DNA (large-sized molecules) was first removed using 0.5× beads; small-sized DNA (linear and circular) was then collected using 1.8× beads. This approach was followed 3 times on each sample for optimal selection outcome. The size-selected tissue DNA was incubated with exonuclease V (New England Biolabs) in a 50 μL reaction system at 37°C for 30 minutes for linear DNA removal. The remaining DNA was collected by column purification using MinElute Reaction Cleanup Kit (QIAGEN), followed by tagmentation or RCA for eccDNA library construction. For tagmentation, DNA samples were processed with Nextera XT DNA Library Preparation Kit (Illumina). For RCA, DNA samples were amplified using NxGen phi29 DNA Polymerase (Lucigen) at 30°C for 12 hours, followed by sonication to 200 bp and sequencing adapter ligation. DNA libraries were sequenced on Illumina NextSeq 500 or NextSeq 2000 platforms as 2 × 75 bp or 2 × 150 bp paired-end reads.

### EccDNA identification and characterization

Details of the bioinformatics principles for mouse eccDNA identification, size profiling, and genomic annotation followed a previous study (4) with minor adjustments, including the use of mouse genomes as reference genomes. In particular, to define the expected frequencies of eccDNA in various genomic elements, we first generated eccDNA loci from random genomic coordinates via computer simulation. We then calculated the frequencies of these artificially generated eccDNA loci across various genomic elements. We denoted these frequency values generated by computer simulation as expected frequencies. For the mouse pregnancy model, mating pairs were set up as follows: female mice of the C57BL/6 genomic background (wild-type or *Dnase1l3*^−/−^) were crossed with male mice from either the BALB/c (wild-type) or the C57BL/6 (*Dnase1l3*^−/−^) genomic background (Supplemental Table 4). Sequencing data of eccDNA libraries from the pregnant mice were first mapped against the C57BL/6 reference genome (National Center for Biotechnology Information build 38/UCSCmm10) for candidate eccDNA identification. The resultant candidate eccDNA reads were subsequently mapped against the BALB/c genome. Only the candidate reads identified as eccDNA under both C57BL/6 and BALB/c genomes were selected for downstream analyses. A database containing 4,576,884 SNPs that differ between the C57BL/6 and the BALB/c genomes was obtained from the Mouse Genomes Project (https://www.sanger.ac.uk/science/data/mouse-genomes-project). Because all female mice were from the C57BL/6 strain, any eccDNA harboring BALB/c-specific alleles were designated as fetus-specific molecules. The remaining molecules covering the same allele with shared SNPs were designated as shared molecules predominantly of maternal origin.

### Data availability

Sequence data generated in this study have been deposited at the European Genome-Phenome Archive (https://ega-archive.org/datasets) hosted by the European Bioinformatics Institute (accession EGAS00001005873).

### Statistics

Kruskal-Wallis test followed by Dunn’s multiple-comparison test was applied to compare 3 groups of data. Wilcoxon’s rank-sum test was applied to compare 2 groups of data. These statistical tests were performed using GraphPad Prism 8.0 (GraphPad Software). Statistical significance was defined as *P* < 0.05.

### Study approval

#### Animal models.

Mice carrying a targeted allele mutation of *Dnase1* [*Dnase1*^tm1.1(KOMP)Vlcg^] of the C57BL/6 (B6) background were obtained from the Knockout Mouse Project Repository of the University of California, Davis; mice with a CRISPR/Cas9-targeted deletion of exon 5 in *Dnase1l3* of the B6 background were generated by The Jackson Laboratory. These mice were acquired under a third-party distribution agreement and are nontransferable. Mice including wild-type B6 and BALB/c strains were maintained in the Laboratory Animal Center of the Chinese University of Hong Kong (CUHK), with all experimental procedures approved by the Animal Experimentation Ethics Committee of CUHK in compliance with the *Guide for the Care and Use of Laboratory Animals* (8th ed., 2011, National Academies Press) established by the NIH.

#### Human patients.

Four healthy human participants were recruited from the Department of Chemical Pathology, the Chinese University of Hong Kong, with written informed consent. Three human participants with *DNASE1L3* mutations were recruited from the Istituto Giannina Gaslini, also with written informed consent. Four blood samples in total were obtained from this patient cohort. Patient P1 was a boy of Albanian origin who presented at 5 years of age with a steroid-resistant nephrotic syndrome. At 7 years of age, a renal biopsy was performed and revealed a membranous glomerulonephritis (stage II). In the same year, progression of disease led to end-stage renal disease (ESRD) and kidney transplant, complicated by graft failure, and subsequent start of hemodialysis treatment, currently ongoing. P1 provided blood samples at both pre- and posthemodialysis time points. Patient P2 was a girl of Italian nationality who presented at 4 years of age with malar rash, diffuse urticarial erythematous rash, nonerosive arthritis, hemolytic anemia, hematuria, and proteinuria. Blood tests showed elevation of inflammation markers, increased creatinine, hypocomplementemia, and antinuclear antibody (ANA) positivity (1:640). A kidney biopsy performed at 12 years of age revealed a class III C lupus nephritis. Despite treatment, the patient progressed to ESRD requiring hemodialysis and at 13 years old a kidney transplant. Patient P3 was an 8-year-old boy of Italian nationality who presented at 3 years of age with diffuse lymph node enlargement, recurrent fever episodes with increased inflammatory markers, arthritis, nonpruritic urticarial like lesions, and at lab exams microcytic anemia and complement consumption. ANA was borderline (1:80). Fas-mediated apoptosis test and CD4^−^CD8^−^ lymphocyte counts were within normal ranges. His renal function and urine tests were within normal ranges. The 3 patients had the following sequence variations in *DNASE1L3*: P1 and P3 had homozygous mutation of c.290_291delCA (p.Thr97Ilefs*2); P2 was heterozygous for c.290_291delCA (p.Thr97Ilefs*2) with a deletion in exon 5. The study was approved by the Joint Chinese University of Hong Kong-Hospital Authority New Territories East Cluster Clinical Research Ethics Committee and the ethics committee of the Istituto Giannina Gaslini.

## Author contributions

STKS, SHC, PJ, KCAC, RWKC, and YMDL designed research; STKS performed experiments; RWYC designed the CRISPR/Cas9-targeted deletion of the *Dnase1l3*^−/−^ mice; RWYC, DKLW, and KOL coordinated the animal work; SV, AV, PF, and PB recruited participants and collected plasma samples from human participants with *DNASE1L3* mutations; STKS, JD, LJ, MY, PJ, KCAC, RWKC, and YMDL analyzed data; and STKS, PJ, KCAC, RWKC, and YMDL wrote the paper. The method used in assigning the authorship order among the 3 co–first authors was as follows: STKS is the project holder; JD performed the majority of bioinformatics analyses; and LJ performed bioinformatics analyses and provided supervision to JD.

## Supplementary Material

Supplemental data

## Version 1 04/22/2022

Electronic publication
