# Supplementary material for: Effects of nucleases on cell-free extrachromosomal circular DNA
Source: JCI Insight. 2022 Apr 22;7(8):e156070. doi: 10.1172/jci.insight.156070 (PMC9089787; doi:10.1172/jci.insight.156070)
Supplement: Supplemental data [file jciinsight-7-156070-s126.pdf]

Supplementary Figure 1: Area-under-the-curve (AUC) values of plasma eccDNA in wild-type ($N = 12$), *Dnase1*$^{-/-}$ ($N = 11$) and *Dnase1l3*$^{-/-}$ ($N = 11$) mice. $P < 0.0001$ for both peak clusters, Kruskal-Wallis test.

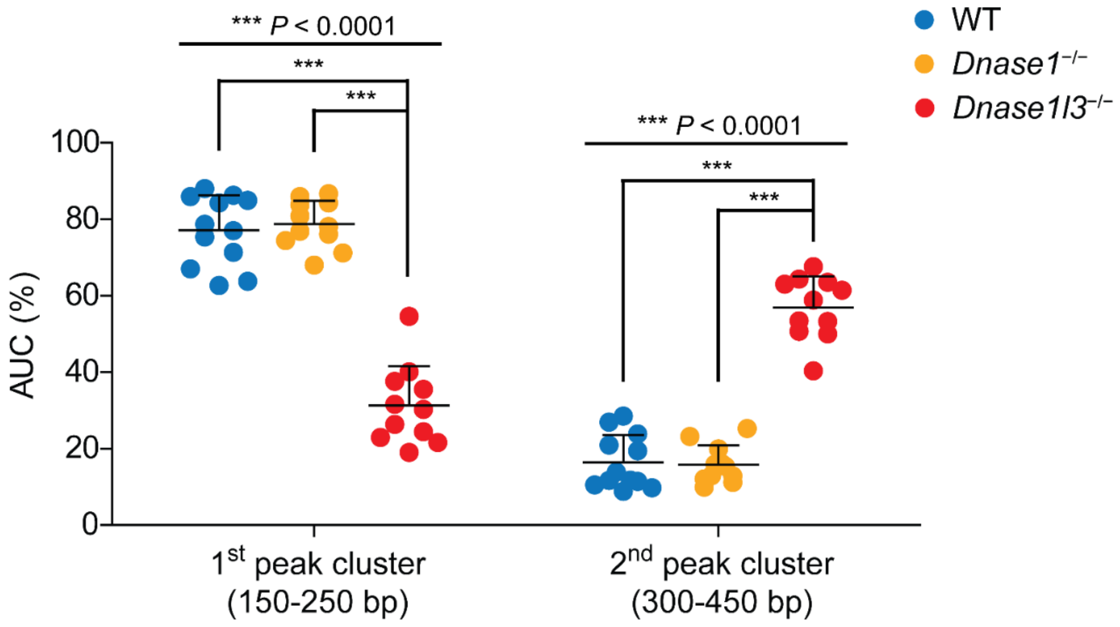

Supplementary Figure 2: EccDNA distributions across various genomic elements relative to the expected frequencies. To define the expected frequencies, we first generated eccDNA loci from random genomic coordinates via computer simulation. We then calculated the frequencies of these artificially-generated eccDNA loci across various genomic elements. These frequency values generated by computer simulation were denoted as expected frequencies. The observed frequencies of eccDNA from real sequencing data were then divided by their expected frequencies for each genomic element, which is denoted as "Fold change" in the y-axis.

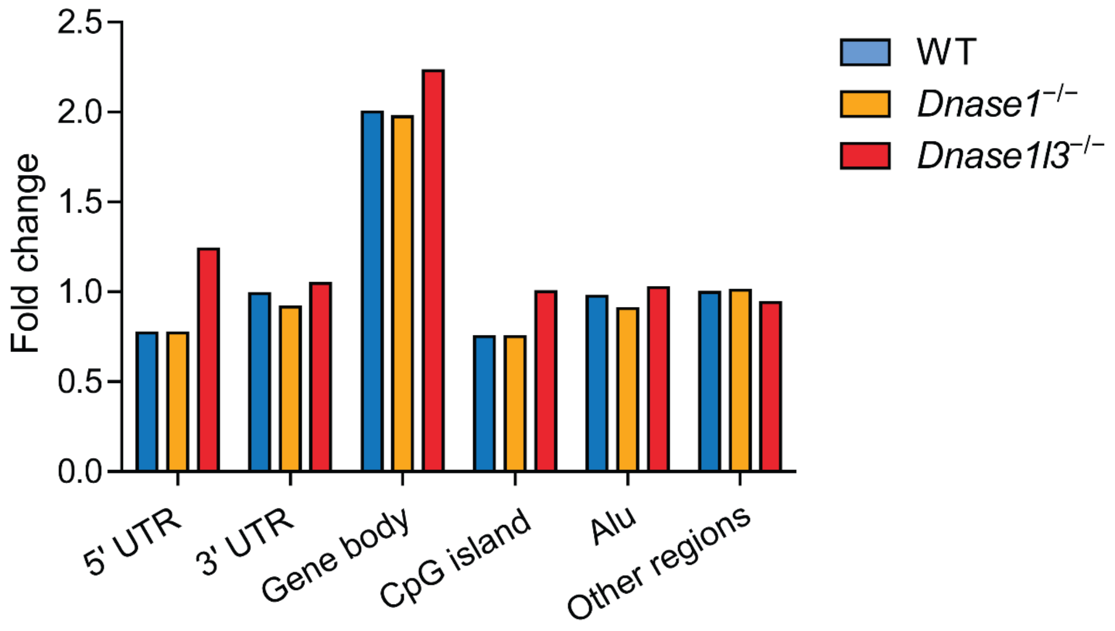

Supplementary Figure 3: Tissue eccDNA identification. (A) Illustrations of experimental approaches for eccDNA enrichment and sequencing from mouse tissues. (B-D) Size profiles of liver eccDNA from wild-type (N = 5), *Dnase1*$^{-/-}$ (N = 5) and *Dnase1l3*$^{-/-}$ (N = 5) mice using the RCA-based method, respectively. (E-G) Size profiles of buffy coat eccDNA from wild-type (N = 6), *Dnase1*$^{-/-}$ (N = 4) and *Dnase1l3*$^{-/-}$ (N = 5) mice using the RCA-based method, respectively.

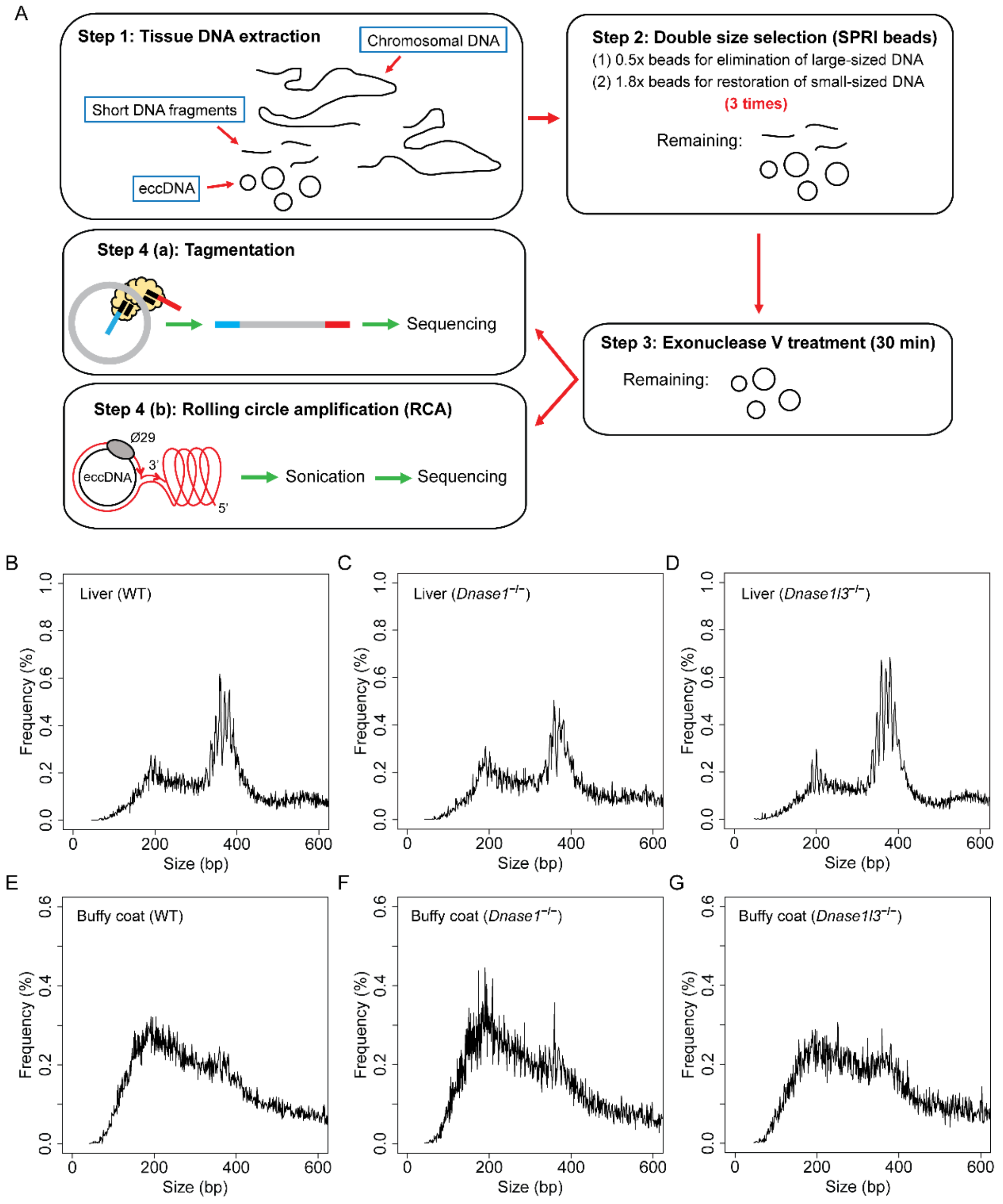

Supplementary Figure 4: Size profiles of tissue eccDNA size in mice. Different colors represent eccDNA size profiles of different individual mice.

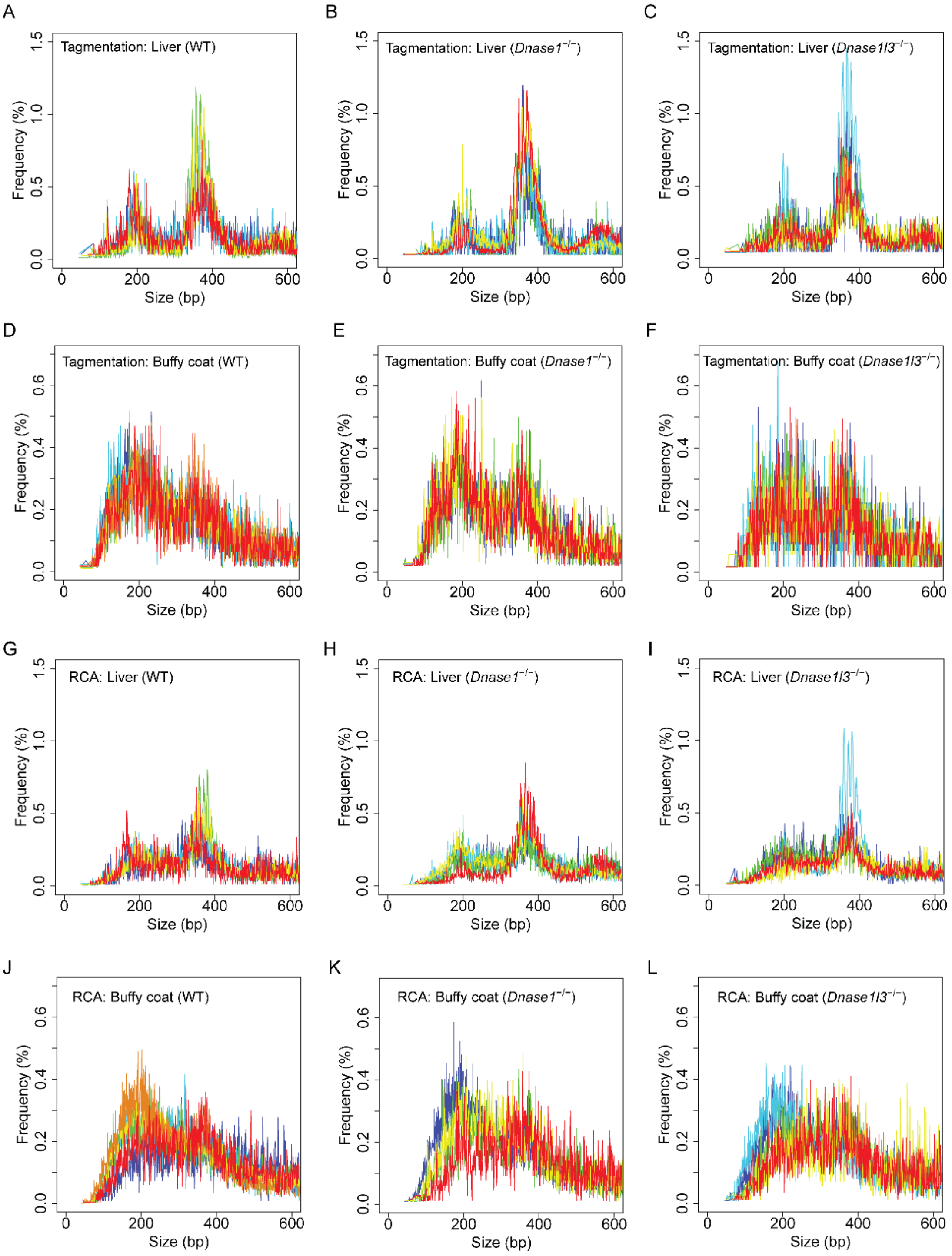

Supplementary Figure 5: Mouse eccDNA size distributions in different tissue types detected by ATAC-seq.

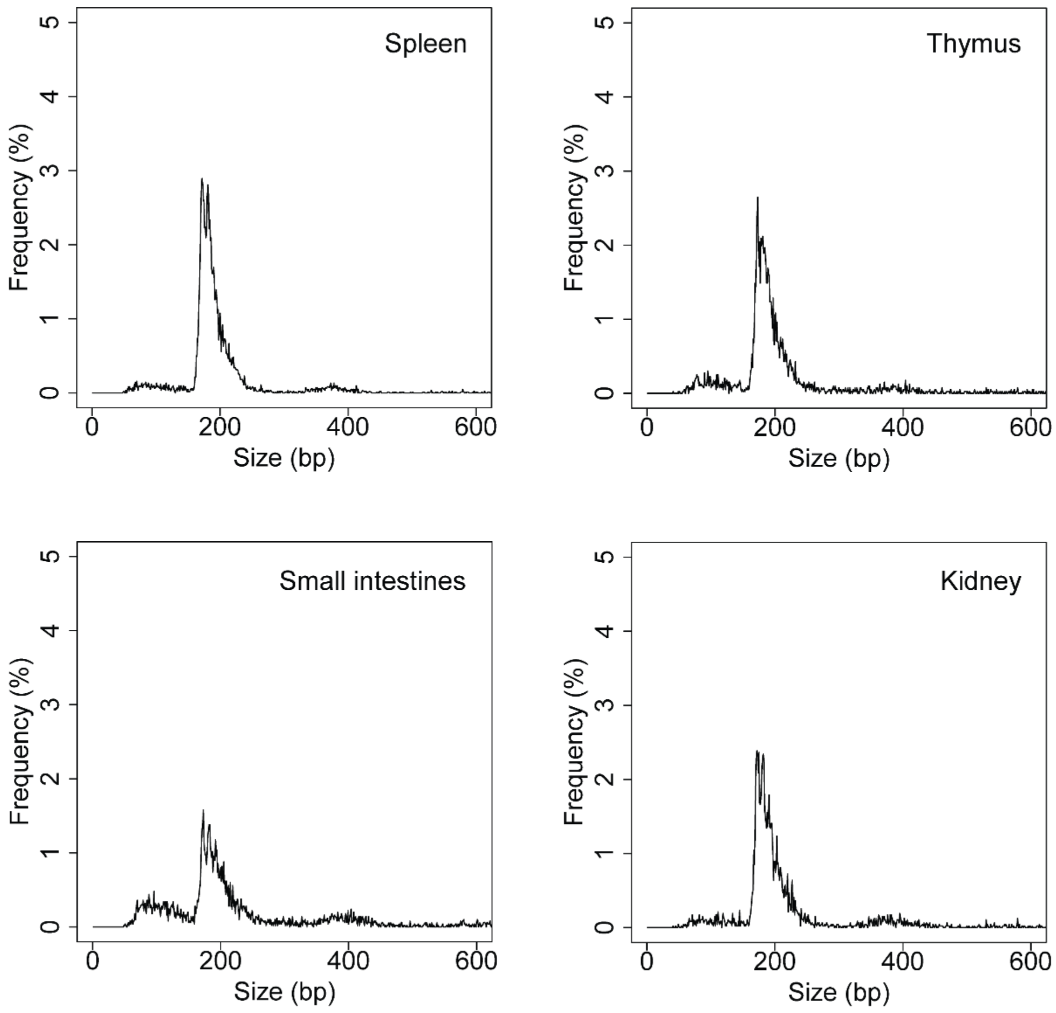

Supplementary Figure 6: Tissue eccDNA size comparisons using the RCA method.

(A) AUC ratios of liver eccDNA of individual mice. *P* = 0.93, Kruskal-Wallis test. (B) AUC ratios of buffy coat eccDNA of individual mice. *P* = 0.93, Kruskal-Wallis test.

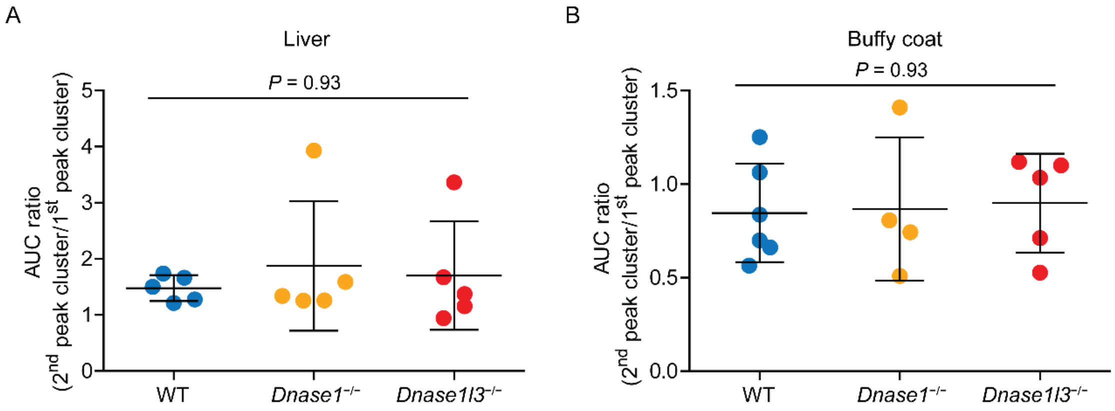

Supplementary Figure 7: Size profiles of (A) shared (predominately maternal-derived) and (B) fetal-derived eccDNA in the plasma of *Dnase1l3*$^{-/-}$ mice carrying *Dnase1l3*$^{+/-}$ fetuses. AUC values of the 1st and 2nd peak clusters are as labelled.

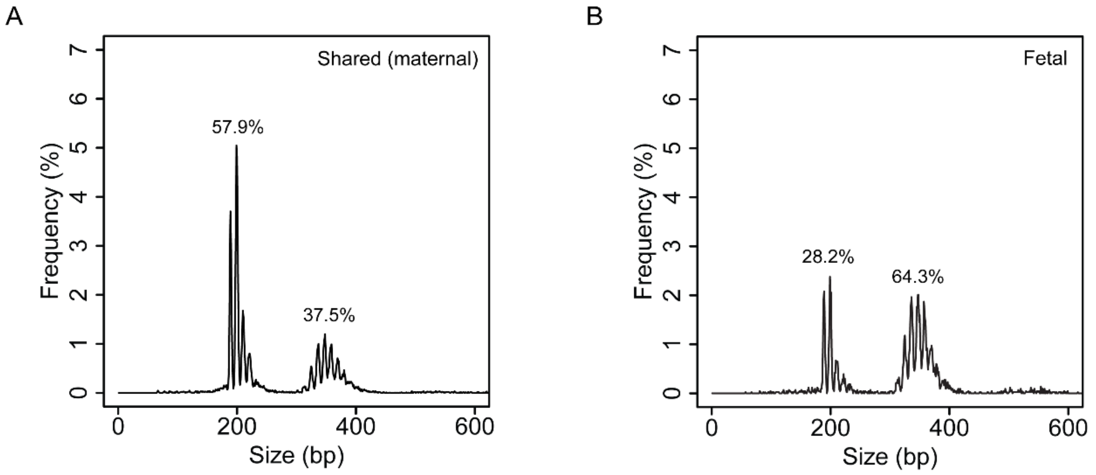

Supplementary Figure 8: Normalized eccDNA counts in human subjects with and without *DNASE1L3* mutations.

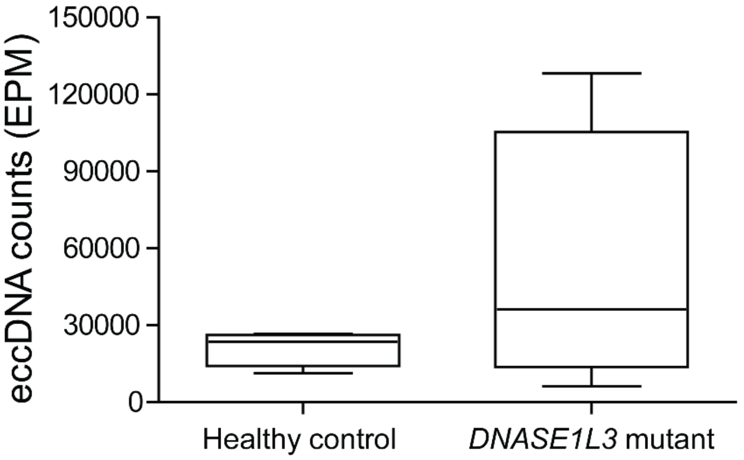

Supplementary Figure 9: EccDNA size profiles of individual plasma samples collected from healthy and *DNASE1L3*-mutated human subjects. HC: healthy control.

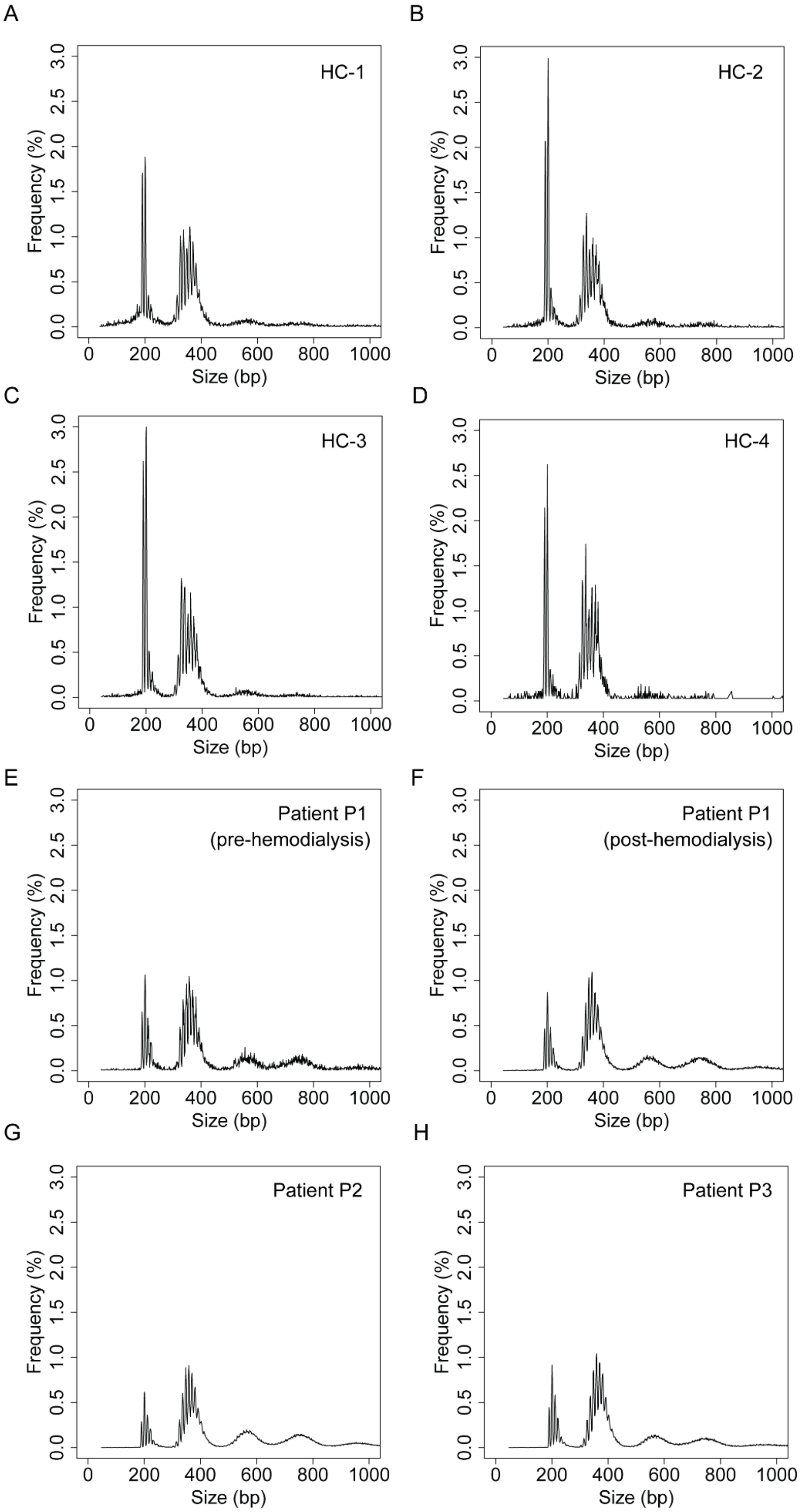

Supplementary Table 1: Sequencing details of mouse plasma DNA samples. EPM: eccDNA per million mappable reads.

Sample	Raw reads	Mapped reads	Mappability (%)	Normalized eccDNA counts (EPM)
WT-1	22,262,301	11,484,399	50.6	2,825
WT-2	20,981,365	14,039,663	67.0	3,742
WT-3	15,805,715	7,069,685	44.7	1,640
WT-4	17,352,765	4,290,827	24.7	2,153
WT-5	12,033,253	6,518,666	54.2	6,468
WT-6	16,411,921	6,187,107	37.7	6,952
WT-7	17,694,509	7,295,671	41.2	4,441
WT-8	15,232,602	6,289,094	41.3	5,381
WT-9	18,092,570	7,785,377	43.0	3,286
WT-10	20,152,676	8,359,660	41.5	1,404
WT-11	16,595,559	7,233,149	43.6	1,826
WT-12	18,925,094	9,484,730	50.1	1,935
Dnase1$^{-/-}$-1	23,354,420	10,848,855	46.5	3,730
Dnase1$^{-/-}$-2	16,161,775	6,004,268	37.2	3,580
Dnase1$^{-/-}$-3	17,573,843	3,549,192	20.2	2,470
Dnase1$^{-/-}$-4	14,607,504	1,745,551	11.9	3,366
Dnase1$^{-/-}$-5	15,764,932	5,490,534	34.8	1,677
Dnase1$^{-/-}$-6	14,462,973	7,327,291	50.7	4,320
Dnase1$^{-/-}$-7	19,795,332	9,730,252	49.2	6,776
Dnase1$^{-/-}$-8	19,611,100	8,597,020	43.8	5,680
Dnase1$^{-/-}$-9	13,896,140	7,162,998	51.5	1,980
Dnase1$^{-/-}$-10	12,409,262	5,359,667	43.2	5,568
Dnase1$^{-/-}$-11	12,666,650	4,752,644	37.5	5,082
Dnase1l3$^{-/-}$-1	19,037,213	3,137,802	16.5	15,830
Dnase1l3$^{-/-}$-2	18,637,739	1,113,037	6.0	12,206
Dnase1l3$^{-/-}$-3	26,555,335	15,005,907	56.5	1,241
Dnase1l3$^{-/-}$-4	27,836,098	4,506,201	16.2	4,033
Dnase1l3$^{-/-}$-5	20,194,544	2,706,402	13.4	14,587
Dnase1l3$^{-/-}$-6	15,084,008	6,516,553	43.2	40,897
Dnase1l3$^{-/-}$-7	11,845,852	4,411,255	37.2	17,825
Dnase1l3$^{-/-}$-8	16,152,517	8,247,827	51.1	5,381
Dnase1l3$^{-/-}$-9	14,648,472	7,680,299	52.4	2,557
Dnase1l3$^{-/-}$-10	15,418,207	6,599,296	42.8	4,119
Dnase1l3$^{-/-}$-11	15,330,036	6,58,3005	42.9	12,615

Supplementary Table 2: Top 5 combinations of trinucleotide motifs flanking the eccDNA junctions in wild-type mice.

I	II	III	IV	Frequency (%)
TGT	GTG	TGT	GTG	0.0403
ACA	CAC	ACA	CAC	0.0356
GTG	TGT	GTG	TGT	0.019
CAC	ACA	CAC	ACA	0.0185
AAA	AAA	AAA	AAA	0.0095

Supplementary Table 3: Top 5 combinations of trinucleotide motifs flanking the eccDNA junctions in *Dnase1l3*$^{-/-}$ mice.

I	II	III	IV	Frequency (%)
TGT	GTG	TGT	GTG	0.0104
ACA	CAC	ACA	CAC	0.0095
CAC	ACA	CAC	ACA	0.0065
GTG	TGT	GTG	TGT	0.003
AAA	CTT	TGA	CTT	0.0027

Supplementary Table 4: Details of mouse pregnancy samples.

Mouse ID	Mating pairs				Maternal age (weeks)	Days of pregnancy	Fetuses			Total eccDNA detected	EccDNA covering informative SNPs		Fetal eccDNA fraction (%)
	Female		Male										
	Dnase1l3 genotype	Strain	*Dnase1l3* genotype	Strain			Number	*Dnase1l3* genotype	Strain		Shared	Fetal-specific	
Mu582	+/+	B6	+/+	BALB/c	12	15	8	+/+	B6, BALB/c	100617	2178	325	26.0%
Mu614					13	19	9			239898	4885	869	30.2%
Mu617					12	19	7			249601	5602	502	16.5%
Mu620					12	16	8			106471	1644	231	24.6%
Mu597	−/−	B6	−/−	B6	13	19	8	−/−	B6	781293	---	---	---
Mu616					13	19	9			242992	---	---	---
Mu587					10	16	7			159028	---	---	---
Mu586	−/−	B6	+/+	BALB/c	12	15	3	+/−	B6, BALB/c	160795	2709	390	25.2%
Mu596					13	18	4			413757	6110	1856	46.6%
Mu606					11	15	9			437321	6670	987	25.8%
Mu615					12	19	3			503316	4367	648	25.8%
Mu618					12	16	3			137275	2404	448	31.4%

Supplementary Table 5: Sequencing details of human plasma samples. HC: plasma samples from healthy control subjects.

Samples	Raw reads	Mapped reads	Mappability (%)	eccDNA loci
HC-1	104,592,793	24,427,974	23.4	23,637
HC-2	216,521,242	10,099,476	4.7	13,456
HC-3	210,078,058	16,000,804	7.6	19,416
HC-4	111,990,276	4,389,800	3.9	3,737
Patient P1 (pre-hemodialysis)	127,603,364	21,262,279	16.7	13,184
Patient P1 (post-hemodialysis)	187,323,954	64,355,668	34.4	81,634
Patient P2	201,156,353	78,998,438	39.3	380,521
Patient P3	181,482,761	116,864,599	64.4	242,735
